# Inter- and intraobserver reliability of morphological Mutch classification for greater tuberosity fractures of the proximal humerus: A comparison of x-ray, two-, and three-dimensional CT imaging

**DOI:** 10.1371/journal.pone.0259646

**Published:** 2021-11-11

**Authors:** Sam Razaeian, Said Askittou, Birgitt Wiese, Dafang Zhang, Afif Harb, Christian Krettek, Nael Hawi

**Affiliations:** 1 Trauma Department, Hannover Medical School, Hannover, Lower Saxony, Germany; 2 Department of Internal Medicine, KRH Klinikum Lehrte, Lehrte, Lower Saxony, Germany; 3 Department of General Medicine, Hannover Medical School, Hannover, Lower Saxony, Germany; 4 Department of Orthopaedic Surgery, Brigham and Women’s Hospital, Boston, Massachusetts, United States of America; University Hospital Zurich, SWITZERLAND

## Abstract

**Background:**

The objective of this study was to investigate inter- and intraobserver reliability of the morphological Mutch classification for greater tuberosity (GT) fragments in consecutive proximal humerus fractures (PHF) regardless of the number of parts according to the Codman classification system for three different imaging modalities (plain radiographs, two-dimensional [2-D] computed tomography [CT], and reformatted, three-dimensional [3-D] CT reconstruction).

**Materials and methods:**

One hundred thirty-eight consecutive PHF with GT involvement were identified between January 2018 and December 2018 in a supraregional Level 1 trauma center. GT morphology was classified by three blinded observers according to the morphological Mutch classification using the picture archiving and communication software Visage 7.1 (Visage Imaging Inc., San Diego, CA, USA). Fleiss’ and Cohens’ kappa were assessed for inter- and intraobserver reliability. Strength of agreement for kappa (k) values was interpreted according to the Landis and Koch benchmark scale.

**Results:**

In cases of isolated GT fractures (n = 24), the morphological Mutch classification achieved consistently substantial values for interobserver reliability (radiograph: k = 0.63; 2-D CT: k = 0.75; 3-D CT: k = 0.77). Moreover, use of advanced imaging (2-D and 3-D CT) tends to increase reliability. Consistently substantial mean values were found for intraobserver agreement (radiograph: Ø k = 0.72; 2-D CT: Ø k = 0.8; 3-D CT: Ø k = 0.76). In cases of multi-part PHF with GT involvement (n = 114), interobserver agreement was only slight to fair regardless of imaging modality (radiograph: k = 0.3; 2-D CT: k = 0.17; 3-D CT: k = 0.05). Intraobserver agreement achieved fair to moderate mean values (radiograph: Ø k = 0.56; 2-D CT: Ø k = 0.61; 3-D CT: Ø k = 0.33).

**Conclusion:**

The morphological Mutch classification remains a reliable classification for isolated GT fractures, even with 2-D or 3-D CT imaging. Usage of these advanced imaging modalities tends to increase interobserver reliability. However, its reliability for multi-part fractures with GT involvement is limited. A simple and reliable classification is missing for this fracture entity.

## Introduction

While there is currently no ideal classification system for proximal humeral fractures (PHF), several have been proposed in order to guide treatment [1]. The most widely used classification systems, in both clinical and research settings, are those of Neer and AO/OTA (Arbeitsgemeinschaft für Osteosynthesefragen/Orthopaedic Trauma Association) [2–6].

However, particularly in cases of greater tuberosity (GT) fractures, their reliabilities are limited, which limits comparisons of outcomes of treatment methods among different clinical trials and reports [1, 2].

In response to this lack of a reliable classification for scientific communication and surgical decision-making, Mutch and colleagues have described a classification of GT fractures that is based on fracture morphology [2]. It separates fractures into three types (avulsion, split, and depression) that are easily identifiable on plain radiographs and performs superiorly to the Neer or AO/OTA classifications for inter- and intraobserver reliability. This morphological Mutch classification has the advantage of being simple, uses the standard radiological views of the shoulder, involves no additional radiation exposure or cost, and has a good to excellent inter- and intraobserver reliability. Furthermore, it might have practical implications in terms of pathophysiology and surgical fixation technique. This classification system is conceptually designed and validated for radiographic evaluations of isolated GT fractures [2].

Recently, a potential application of this classification system to PHF with GT involvement in general has been discussed, but to date, a reliability analysis for this fracture entity has not been performed [7].

This study aims to investigate inter- and intraobserver reliability of the morphological Mutch classification for GT fragments in consecutive PHF regardless of the number of parts according to Codman’s classification system for three different imaging modalities (plain radiographs, two-dimensional [2-D] computed tomography [CT], and reformatted, three-dimensional [3-D] CT reconstruction).

## Materials and methods

### Patients

This study consists of consecutive cases of PHF from an observational registry study (Hannover Humerus Registry–HHR). HHR is a prospective, CT-based single center registry study of a supraregional Level 1 trauma center, aiming to investigate the healing process of PHF and humeral shaft fractures. A primary early function conservative treatment regimen is provided to all patients with PHF, except in cases of locked fracture-dislocations, head split fractures, open fractures, concomitant vascular injury, or patient request for surgery. There are no cut-offs for the conservative treatment regimen in this ongoing observational registry study, including age, amount of displacement in millimeters or centimeters, and the degree of coronal or sagittal fracture angulation. All patients older than 18 years, except pregnant women, admitted to the emergency department obtain a CT of the proximal humerus in addition to conventional radiographs (AP and scapular-Y views). The study is authorized by the local ethical committee (journalno. 322–2016). The protocol is registered at ClinicalTrials.gov (NCT03060876). All patients gave written consent.

Between January 2018 and December 2018, 225 consecutive cases were registered and could be retrospectively examined CT-based for this study. All fractures were classified by one fellowship-trained senior physician with special focus on upper extremity surgery (N.H.) according to the Codman and Neer classification system [8, 9]. Every fracture with a GT fragment that had more than two parts according to the Codman classification system was defined as a multi-part PHF with GT involvement. Humeral shaft fractures (22) and pediatric fractures (5), fractures without GT involvement (47), and records with incomplete datasets (13) were excluded. A final cohort of 138 PHF with GT involvement according to the Codman classification system were available for the analysis. These included 24 (17.4%) isolated GT fractures. Among the remaining multi-part PHF with GT involvement, the following fracture types according to the Neer classification system were identified: 64 (56.1%) I, 1 (0.9%) II, 21 (18.4%) III, 26 (22.8%) IV, and 2 (1.8%) VI.

**Fig 1:**
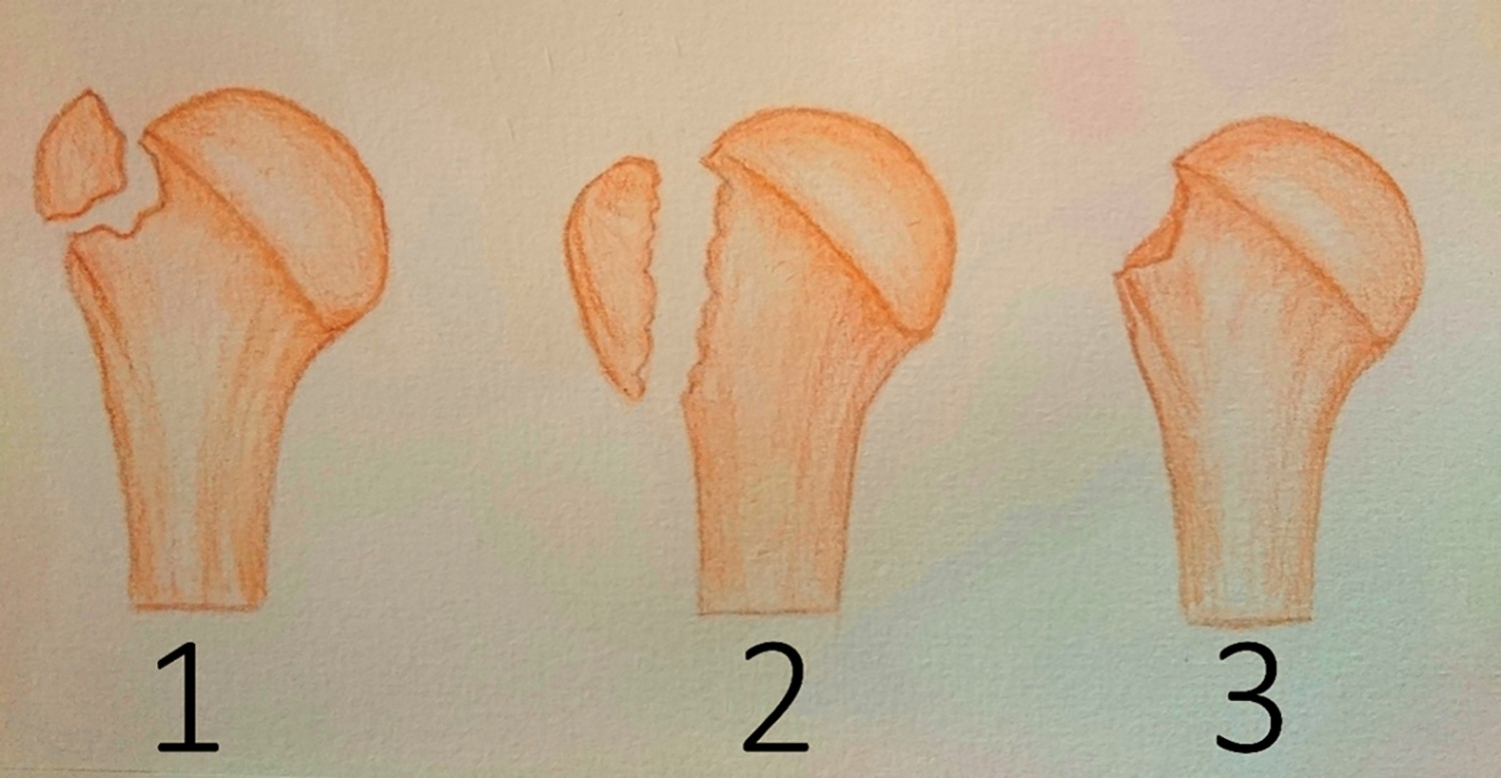
Morphological Mutch classification system. Illustration shows the three types of the morphological Mutch classification (type 1: avulsion, type 2: split, type 3: depression).

Three observers with different levels of experience classified the records in a blinded and randomized fashion according to the morphological Mutch classification (Fig 1) with the picture archiving and communication software Visage 7.1 (Visage Imaging Inc., San Diego, CA, United States) [2].

The group of observers consisted of one senior resident of orthopedic trauma surgery (A.H.), one senior resident of orthopedic trauma surgery trained in classification systems of PHF (S.R.), and one untrained, non-specialist resident (S.A.).

**Fig 2:**
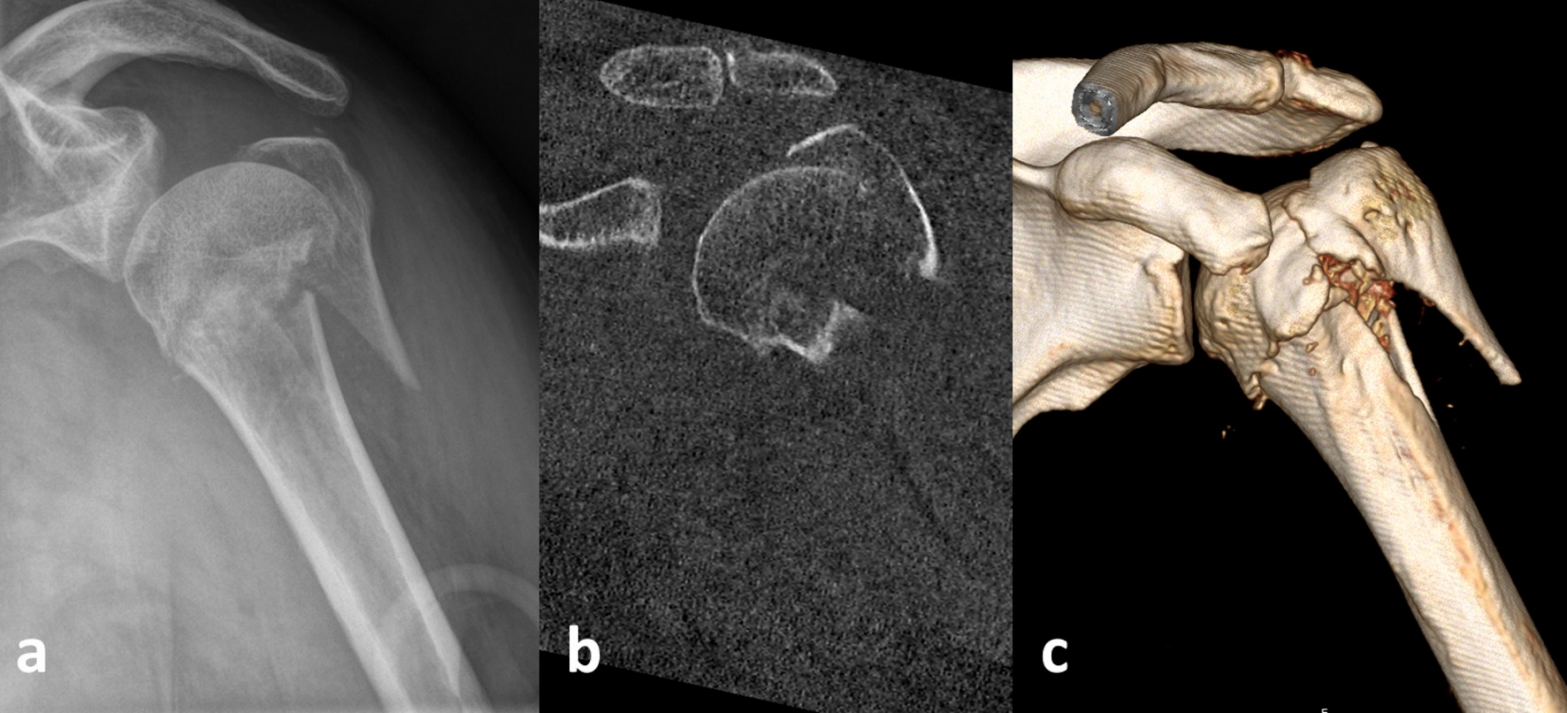
Split type 2 of GT fragment according to the morphological Mutch classification in a multi-part fracture of a 62-year-old patient. Plain radiograph (a), 2-D CT (b), and 3-D CT (c).

Examination of the records was performed in isolation for each imaging modality (plain radiographs, 2-D and 3-D CT) (Fig 2).

At a minimum of three months after completion of the initial review process, all radiographs and CT scans were reevaluated, and data collection proceeded a second time in the same fashion to allow for intraobserver reliability analysis.

Variables including age and gender were also collected and analyzed.

### Statistical analyses

Descriptive statistics, including means, standard deviations, and ranges were calculated. To assess inter- and intraobserver reliability Fleiss’ and Cohens’ kappa were determined [10, 11]. The Landis and Koch benchmark scale was used to interpret the strength of agreement for kappa (k) values [12].

According to Landis and Koch, kappa coefficients <0 indicate no agreement; 0.0 to 0.2, slight agreement; 0.21 to 0.4, fair agreement; 0.41 to 0.6, moderate agreement; 0.61 to 0.8, substantial agreement; and 0.81 to 1.0, almost perfect agreement. For the analyses, SPSS 26 (IBM, Armonk, New York) and Microsoft Excel 2016 (Microsoft Corporation, Redmond, Washington) were used.

## Results

**Fig 3:**
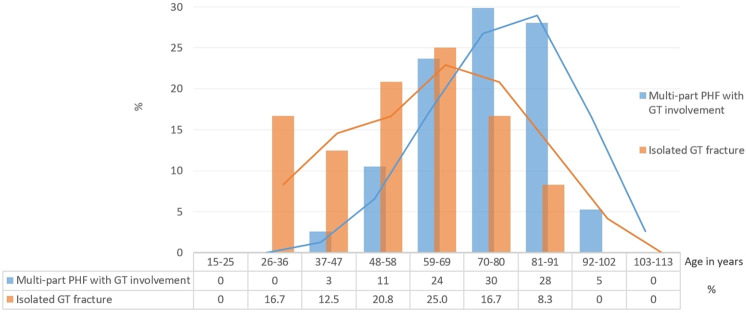
Histogram of both examined fracture groups showing age distribution and moving average trend lines.

Ninety-six (69.6%) patients were female and 42 (30.4%) were male. The average age was 70.6 years (range, 26 to 97 years). Fig 3 shows the age distribution of both types of fracture groups in histograms.

In cases of isolated GT fractures (n = 24), the morphological Mutch classification achieved consistently substantial values for interobserver reliability. The usage of advanced imaging (2-D and 3-D CT) tended to increase kappa values. Consistently substantial mean values were achieved for intraobserver agreement (Tables 1 and 2).

**Table 1:** Interobserver reliability assessed with Fleiss’ kappa (k).

Fracture entity	Imaging modality	Fleiss’ kappa (k)
**Multi-part fracture with GT involvement**	***Radiograph***	0.3
	***2-D CT***	0.17
	***3-D CT***	0.05
**Isolated GT fracture**	***Radiograph***	0.63
	***2-D CT***	0.75
	***3-D CT***	0.77

**Table 2:** Intraobserver reliability assessed with Cohens’ kappa (k).

Fracture entity	Imaging modality	Cohens‘ kappa (k)^a^	Range
**Multi-part fracture with GT involvement**	***Radiograph***	0.56	0.34 to 0.83
	***2-D CT***	0.61	0.24 to 1
	***3-D CT***	0.33	0 to 0.74
**Isolated GT fracture**	***Radiograph***	0.72	0.7 to 0.85
	***2-D CT***	0.8	0.54 to 1
	***3-D CT***	0.76	0.65 to 0.85

^a^Kappa values are given as mean values.

In cases of multi-part PHF with GT involvement (n = 114), interobserver agreement was only slight to fair, regardless of imaging modality. Mean values for intraobserver agreement in this group were fair to moderate (Tables 1 and 2).

Table 3 provides details of observers`selections for both fracture groups as well as each imaging modality. Avulsion type was the predominant fracture pattern among isolated GT fractures, while split type was the predominant fracture pattern among multi-part fractures.

**Table 3:** Total amount of observers`selections (n (%)) for both fracture groups, and each imaging modality.

Fracture entity	Imaging modality	Morphological Mutch type		
		*Avulsion type*	*Split type*	*Depression type*
**Multi-part fracture with GT involvement**	***Radiograph***	94 (13.74)	580 (84.8)	10 (1.46)
	***2-D CT***	36 (5.26)	646 (94.44)	2 (0.29)
	***3-D CT***	31 (4.53)	650 (95.03)	3 (0.44)
**Isolated GT fracture**	***Radiograph***	81 (56.25)	49 (34.03)	14 (9.72)
	***2-D CT***	74 (51.39)	57 (39.58)	13 (9.03)
	***3-D CT***	75 (52.08)	54 (37.5)	15 (10.42)

Total amount consists of double-time selections of three observers.

## Discussion

### Principal findings

This is the first study investigating inter- and intraobserver reliability of the morphological Mutch classification for GT fragments in consecutive PHF regardless of the number of parts according to Codman classification system.

The study findings confirm the morphological Mutch classification as a simple, reliable classification system for isolated GT fractures, regardless of the observer’s level of experience, and including with the use of advanced imaging (2-D and 3-D CT). In addition, the findings reveal that the use of these advanced imaging modalities tends to increase reliability, although this tendency is only noticeable when considering the quantitative data rather than the categorical interpretation of strength of agreement. To the best of our knowledge, this is the first study investigating the reliability of the morphological Mutch classification for advanced imaging.

In the group of multi-part PHF with GT involvement, the reliability of the Mutch classification was limited regardless of imaging modality. This might indicate a continued lack of a simple and reliable classification system that helps inform the prognosis or treatment implications of the various morphologies found in these fractures, whereby it should be noted that the Mutch classification system was not intended to be applicable for this type of fracture, but originally designed and validated only for isolated GT fractures [7].

### Strengths and limitations

The findings of this study are based on several strengths. These include the consecutive inclusion of all patients with PHF at a supraregional Level 1 trauma center as well as the consistent and comprehensive diagnostic imaging of all fractures regardless of fracture pattern or treatment regimen as part of an ongoing observational registry study. Therefore, a wide variety of fracture patterns was included and analyzed in contrast to previous studies, which selected more severe fracture configurations with surgical implication [7].

In addition, the intentional inclusion and analysis of isolated GT fractures was not only used as a confirmation of previously reported findings, but also served also as an internal control of observers`rating quality using this classification.

Nevertheless, this study has several limitations to consider.

Firstly, this study included only a low number of observers. It is unclear that additional observers would have influenced the findings significantly, especially in the group with multi-part fractures with GT involvement.

Secondly, all imaging modalities were used in isolation for classification rather than using plain radiography in combination with 2-D and 3-D CT imaging. It is possible that the inter- and intraobserver agreement would have been higher if all modalities had been used together, thus producing an additive effect. However, the analysis performed in this study involved the evaluation of radiographs, 2-D CT scans, and 3-D CT scans as singular entities. This strategy does not mirror real clinical practice, where surgeons would use all images available in combination for evaluation [3].

Thirdly, while Mutch et al. reviewed 199 cases of isolated GT fractures over a five-year period, the small number of only 24 cases in this study should be considered as a clear limitation. In addition, the colleagues created and validated this classification system for isolated GT fractures. It is therefore not surprising that usage of this classification system for GT fragments in multi-part proximal humerus fractures is not suitable concerning the limited inter- and intraobserver reliability.

Lastly, as is known, the vast majority of PHF occur in patients over the age of sixty years, with the greatest reported incidence among individuals eighty years of age or older [1, 13]. This expected age distribution holds true for the examined subgroup of fractures with GT involvement (Fig 3). This population represents a patient group with a very heterogeneous functional demand for which treatment based on fracture pattern or age alone may not lead to the optimal functional outcomes. Treatment might be commonly based on factors that may be subjective and difficult to quantify [1]. Therefore, the suggestion that effective treatment would begin with appropriate classification of fracture pattern as well as the indispensable need for another classification system expressed by some authors should be questioned, in particular when growing concerns indicate that a plethora of classifications with limited reliability are one of the main causes for the current lack of consensus and substantial variability in the treatment of PHF [1, 3, 7].

Due to limited reliability between surgeons for classifications of PHF, some authors have proposed the use of advanced imaging to provide more detailed fracture analysis and classification [3]. One of the principal findings of this study, that advanced imaging tends to increase the reliability of classification in cases of isolated GT fractures, supports this proposition. However, such an interpretation should be reviewed critically. Even if advanced imaging increases agreement on fracture morphology, it remains unclear if it provides additional information that might change decision-making regarding treatment. This point has been already highlighted by several authors [3, 14]. Specifically, in cases of isolated GT fractures, recent findings have indicated that 2-D or 3-D CT images in addition to radiographs did not influence interobserver agreement of GT fracture characteristic assessment, nor did the addition of these images influence the recommendation for surgical treatment. Rather, surgeons felt slightly more confident about their treatment recommendation when using additional CT images. Therefore, the authors challenged the additional value of CT scans for assessment of GT fractures, especially considering the extra economic burden and radiation exposure [14].

### Future perspective

Recently, a potential application of the Mutch classification on complex PHF with GT involvement (Neer 3- and 4- part) has been explored and discussed with respect to the technical aspects of surgical management. Although 43 typical split and 19 avulsion type fragments could be identified out of 71 examined fractures and the morphology of avulsion type fragments seemed to be distinguishable from that of split type fragments, the authors stated that not all of them could be classified into the three Mutch classification types. Furthermore, a depression type could not be identified in even a single case, contrary to our study (Table 3) [7].

Therefore, an extension of the Mutch classification was proposed to categorize the morphology and location of GT fragments to help inform the technical aspects of surgical management. This extended classification system consists of five types: anterior-split, posterior-split, complete-split, anterior-avulsion, and posterior-avulsion [7].

Whether this more comprehensive classification is reliable and can inform prognosis or the technical aspects of surgical management remains unknown, is not answered with this study, and might be considered in further investigations.

### Conclusion

The morphological Mutch classification remains a reliable classification for isolated GT fractures, even for 2-D or 3-D CT imaging. Usage of these advanced imaging modalities tends to increase interobserver reliability. However, its reliability for multi-part fractures with GT involvement is limited. A simple and reliable classification is lacking for this fracture entity.

## Supporting information

S1 File HHR-study protocol. (PDF)

S2 File Informed consent form-version 1.0. (PDF)
