# Supplementary material for: Inter- and intraobserver reliability of morphological Mutch classification for greater tuberosity fractures of the proximal humerus: A comparison of x-ray, two-, and three-dimensional CT imaging
Source: PLoS One. 2021 Nov 11;16(11):e0259646. doi: 10.1371/journal.pone.0259646 (PMC8584785; doi:10.1371/journal.pone.0259646)
Supplement: S1 File — (PDF) [file pone.0259646.s001.pdf]

Hannover Humerus Registry (HHR)

Responsible Party

Hannover Medical School, Trauma Department

Carl-Neuberg-Street 1, 30625 Hannover, Germany

Sponsor

Hannover Medical School, Carl-Neuberg-Street 1, 30625 Hannover, Germany

Collaborator

Traumastiftung gGmbH Foundation, Hannover Medical School, Carl-Neuberg-Street 1, 30625 Hannover, Germany

Information provided by (Responsible Party)

Clinical trial registration

ClinicalTrials.gov Identifier NCT03060876

Protocol version

February 23, 2017

Principal investigator

Professor Doctor Christian Krettek, MD, FRACS, FRCSEd

Hannover Medical School, Trauma Department, Carl-Neuberg-Street 1, 30625 Hannover, Germany

Phone: 0049 511 532 2050

Investigators / Research physician

Professor Doctor Nael Hawi, MD, MBA

Hannover Medical School, Trauma Department, Carl-Neuberg-Street 1, 30625 Hannover, Germany

Phone: 0049 511 532 2099

Study nurses

Mrs. Marthe Greeske

Mrs. Katja Ahrens

Email: UCH.TeamHHR@mh-hannover.de

Phone: 0049 511 532 8427

Funding

The study is funded by the aboved-mentioned sponsor and collaborator. These funding sources have no role in the design of this study and will not have any role during its execution, analyses, interpretation of the data, or dissemination of results in the form of publication.

Study Description

This register study collects data of patients with proximal humerus and humerus shaft fractures to monitor the healing process by assessing shoulder function and quality of life according to the treatment method (operative or conservative management).

Detailed Description

Proximal humeral fractures are a common injury, representing about 6% of all adult fractures. Around 70% of these fractures occur in patients over the age of 60, with the greatest reported incidence among people aged 80 or older. The incidence of proximal humeral fractures has been increasing over the past few decades owing to an ageing population and the associated increase in osteoporosis and low energy falls from standing height. The latest Cochrane review suggests that non-operative management might have similar functional outcomes to operative management with lower risks of complications and reoperation, but there is insufficient evidence from current randomised controlled trials to inform decision making between different non-surgical, surgical, or rehabilitation interventions for these fractures.

This prospective data collection of patients with proximal humerus and humerus shaft fractures in a lifelong aftercare treatment plan is for analyzing the healing process in one patient and compared in the whole cohort.

According to actual scientific and clinical issues can this data collection be a basis in optimizing the treatment method.

Investigated condition or disease

Proximal humerus fractures

Humeral shaft fractures

Study Design

Study model: Prospective, observational, non-interventional registry study

Study setting: Monocentric study (Supraregional Level 1 trauma center in Germany)

Estimated enrollment: 1000 participants

Observational model: Cohort

Time perspective: Prospective

Target Follow-Up duration: 5 years

Actual study start date: June 2016

Estimated primary completion date: June 2021

Estimated study completion date: June 2025

Eligibility Criteria

Ages Eligible for Study: Child, Adult, Older Adult

Sexes Eligible for Study: All

Accepts Healthy Volunteers: No

Sampling Method: Non-Probability Sample

Study Population: Patients with proximal humerus fracture and humeral shaft fractures

Inclusion Criteria

All patients with trauma-related proximal humerus and humerus shaft fractures cognitively able to fill out a questionnaire and to sign informed consent

Exclusion Criteria

All patients with oncological-related proximal humerus and humerus shaft fracture, and cognitively not able to fill out a questionnaire

No informed consent

Outcome Measures

Primary Outcome Measures:

1. Constant Shoulder Score (CS) (change over time) [Time Frame: following a aftercare treatment plan with follow ups after 6,12, 24, 52 weeks in the first year; then through study completion, an average of 1 year]

The CS is a commonly used outcome measure for assessing the outcomes of the treatment of shoulder disorders including the pain score, functional assessment, range of motion and strength measures.

In week 6, 52 and in the yearly follow ups for comparison reasons the investigators also assess the CS of the not affected shoulder. Reported value is the impaired shoulder-functionality in percent. 100% are equivalent to an unimpaired shoulder function.

2. DASH (change over time) [Time Frame: following a aftercare treatment plan with follow ups after 1,3,6,12, 24, 52 weeks in the first year; then through study completion, an average of 1 year]

The Disabilities of the Arm, Shoulder and Hand (DASH) scoring system was developed to assess the level of disability for any patient with any condition affecting the upper limb by covering domains including symptoms, physical function, social function and psychological function.

3. Health related quality of life: EuroQoL (EQ-5D-3L+5L) (change over time)
[Time Frame: following a aftercare treatment plan with follow ups after 1,3,6,12, 24, 52 weeks in the first year; then through study completion, an average of 1 year]

The EQ-5D-3L/5L is a standardized instrument for use as a measure of health outcome. It was designed for self-completion covering mobility, self-care, usual activities, pain/discomfort, and anxiety/depression. Furthermore, health state is indicated by a Visual Analog Scale (VAS). EQ-5D assesses the health status of the patients in an one-dimensional number (1-3/1-5).

4. Subjective Shoulder Value (SSV) (change over time) [Time Frame: following a aftercare treatment plan with follow ups after 1,3,6,12, 24, 52 weeks in the first year; then through study completion, an average of 1 year]

The SSV is defined as a patient's subjective shoulder assessment expressed as a percentage of an entirely normal shoulder, which would score 100%.

Secondary Outcome Measures:

1. Healing process (change over time) [Time Frame: following a aftercare treatment plan with follow ups after 1, 3, 6,12, 24, 52 weeks in the first year; then through study completion, an average of 1 year]

Analyzing the radiological imaging for possible dislocation of fracture fragments or other complications

2. General health conditions [Time Frame: Baseline week 1]

Prior disorders of the affected shoulder and secondary diagnosis which are relevant for healing process of the proximal humerus fracture.

Ethics

This study is authorized by the local ethical committee (Hannover Medical School) (Journalno. 322-2016) and is carried out in accordance with the Ethical standards of the 1964 Declaration of Helsinki as updated in 2004. All patients will give written informed consent obtained by trained official study nurses.

The protocol and the template informed consent forms contained in Appendix I are reviewed and approved by the sponsor and the applicable IRBs/ECs with respect to scientific content and compliance with applicable research and human subjects regulations.

The protocol, site-specific informed consent forms (local language), participant education and recruitment materials, and other requested documents — and any subsequent modifications — also will be reviewed and approved by the ethical review bodies (IRBs/ECs).

Any modifications to the protocol which may impact on the conduct of the study, potential benefit of the patient or may affect patient safety, including changes of study objectives, study design, patient population, sample sizes, study procedures, or significant administrative aspects will require a formal amendment to the protocol. Such amendment will be approved by the Ethics Committee/IRB prior to implementation and notified to the health authorities in accordance with local regulations.

Administrative changes of the protocol are minor corrections and/or clarifications that have no effect on the way the study is to be conducted. These administrative changes will be documented in a memorandum.

Informed consent

Trained study nurses will introduce the trial to patients regarding the main aspects of the trial. Patients will also receive information sheets. Research Nurses will discuss the trial with patients in light of the information provided by information sheets. Patients will then be able to have an informed discussion with the participating consultant. Research Nurses will obtain written consent from patients willing to participate in the trial. Information sheets and consent forms are provided for all parents involved in the trial however these have been amended accordingly in order to provide separate information sheets.

Confidentiality

All study-related information will be stored securely at the study site. All participant information will be stored in locked file cabinets in areas with limited access. All reports, data collection, process, and administrative forms will be identified by a coded ID number only to maintain participant confidentiality. All records that contain names or other personal identifiers, such as locator forms and informed consent forms, will be stored separately from study records identified by code number. All local databases will be secured with password-protected access systems. Forms, lists, logbooks, appointment books, and any other listings that link participant ID numbers to other identifying information will be stored in a separate, locked file in an area with limited access.

Participants' study information will not be released outside of the study without the written permission of the participant-

Data access

All Principal Investigators will be given access to the cleaned data sets. Project data sets will be housed on the Project Accept Web site and/or the file transfer protocol site created for the study, and all data sets will be password protected. Project Principal Investigators will have direct access to their own site's data sets, and will have access to other sites data by request. To ensure confidentiality, data dispersed to project team members will be blinded of any identifying participant information.

Declaration of interests

The investigators, study nurses, their immediate families, and any research foundations with which they are affiliated do not receive any financial payments or other benefits from any commercial entity, and do not have any competing interests related to the subject of this study.
