# Supplementary material for: Inter- and intraobserver reliability of morphological Mutch classification for greater tuberosity fractures of the proximal humerus: A comparison of x-ray, two-, and three-dimensional CT imaging
Source: PLoS One. 2021 Nov 11;16(11):e0259646. doi: 10.1371/journal.pone.0259646 (PMC8584785; doi:10.1371/journal.pone.0259646)
Supplement: S2 File — (PDF) [file pone.0259646.s002.pdf]

MHH Medizinische Hochschule Hannover

UNFALL CHIRURGIE ... immer für Sie da

TRAUMA STIFTUNG

1. Patient/-inneninformation Hannover Humerus Register (HHR)

Erfassung des Heilungsverlaufes, der Lebensqualität und der Funktion nach proximaler Humerusfraktur (= Bruch des schulternahen Oberarmknochens) in Abhängigkeit von der Behandlungsmethode

Die proximale Humerusfraktur ist eine der häufigsten Frakturen des Menschen. Sie tritt überwiegend bei Personen höheren Alters (über 60 Jahre alt) auf.

Eine proximale Humerusfraktur kann sowohl konservativ als auch operativ behandelt werden. Dabei reicht die Spannweite der Verfahren von einem Verband zur Ruhigstellung des Armes über die Fixierung der Knochenfragmente mit Drähten, Schrauben oder Platten bis hin zum Ersatz des Oberarmkopfes durch eine Prothese. Entscheidungskriterien für die Wahl der Behandlungsmethode sind v. a. das Alter und bestehende Vorerkrankungen der Person, die Anzahl und Verschiebung der Knochenfragmente sowie andere Einflüsse.

In der wissenschaftlichen Literatur gibt es keine ausreichenden Aussagen, ob und in welchen Fällen ein bestimmtes Behandlungsvorgehen dem jeweils anderen überlegen ist. Die Wiedererlangung der Funktionsfähigkeit von Schulter und Oberarm ist mit verschiedenen Verfahren (konservativ, OP) möglich. Allerdings haben diese auch unterschiedliche Komplikationen.

Die Klinik für Unfallchirurgie der MHH zielt mit dieser Erfassung im Hannover Humerus Register darauf ab, zu bestimmten Zeitpunkten Daten über den Heilungsverlauf, die Lebensqualität und die Schulter-Arm-Funktion unterschiedlicher Patient/-innen mit verschiedenen Behandlungsmethoden zu erhalten. Diese Informationen werden im Rahmen der normalen medizinischen Behandlung und Verlaufskontrolle erfasst und dann nach diversen Gesichtspunkten ausgewertet. Das Betreuungsangebot für die betroffene Schulter besteht lebenslang. So werden neue wissenschaftliche Erkenntnisse erlangt, beispielsweise welche Behandlungsmethode bei welcher Patient/-innengruppe zu einem optimalen Heilungsverlauf des Oberarmbruches führt (z. B. je nach Altersgruppe, Frakturtyp, bestehenden Risikofaktoren u. v. m.).

Diese Register-Erfassung trägt somit zur stetigen Weiterentwicklung der Behandlungsqualität von Patient/-innen mit proximaler Humerusfraktur unter Berücksichtigung des medizinischen Fortschrittes bei. Um zuverlässige und aussagekräftige Ergebnisse zu bekommen, ist es notwendig eine möglichst hohe Teilnehmendenzahl zu erreichen. Deshalb würden wir gerne die im Rahmen Ihrer (Nach-) Behandlung gewonnenen Daten in verschlüsselter Form (=pseudonymisiert, d. h. Ihr Name wird durch eine Kennnummer ersetzt ohne Angabe von Namen, Anschrift, Initialen o. ä.) im Register erfassen und bitten Sie um Ihre Einwilligung. Die im Rahmen Ihrer Teilnahme an diesem Vorhaben zu erfassenden personenbezogenen Daten werden selbstverständlich vertraulich behandelt.

Die Teilnahme am Register hat für Sie den Vorteil, dass die ärztliche Behandlung und Beratung über einen langen Zeitraum (mind. 2 Jahre, siehe Übersichtsplan Verlaufsbeobachtung) erfolgt, sowie ein lebenslanges Betreuungsangebot für Ihre betroffene Schulter besteht.

Eine Teilnahme an dieser Datenerfassung ist natürlich freiwillig. Die Ablehnung oder der Abbruch hat selbstverständlich keine Nachteile für Ihre weitere medizinische Betreuung.

Kontaktadresse der Registerstelle:
Klinik für Unfallchirurgie der MHH
Studienkoordination Traumastiftung
Carl-Neuberg-Straße 1
30625 Hannover
Tel: + 49 511 532 8427
Fax: + 49 511 532 5877
E-Mail: HHR@mh-hannover.de
FR. GREESKE / FR. ARENS

Kontaktadresse der Klinik:
Klinik für Unfallchirurgie der MHH
Sekretariat Prof. Dr. med. Christian Krettek
Carl-Neuberg-Straße 1
30625 Hannover
Tel: + 49 511 532 2050
Fax: + 49 511 532 5877
E-Mail: info@mhh-unfallchirurgie.de

Übersichtsplan der Verlaufsbeobachtung im Rahmen des Hannover Humerus Registers

Zeitpunkt der Fraktur (Unfall- bzw. Frakturdatum)

Kontrolluntersuchung bis spätestens 1 Woche nach Frakturdatum

- Klinische Untersuchung (Ärztliche Behandlung und/ oder Beratung)
- Bildgebende Verfahren nach klinischem Standard der UCH (Rö/CT) / bei medizin. Notwendigkeit
- Erfassung patient/-innenbezogener und medizinischer Angaben
  (allgemeine Angaben,
  Angaben zum Unfall, zur Fraktur sowie ggf. zu Begleitverletzungen,
  Angaben zur (Nach-) Behandlung der Fraktur,
  Angaben zum Zustand vor der Fraktur)
- Ausfüllung von Fragebögen
  (DASH-Score zur Erhebung von Einschränkungen in Arm, Schulter und Hand,
  EQ-5D-Fragebogen zur Erhebung der gesundheitsbezogenen Lebensqualität)

Kontrolluntersuchung bis spätestens 3 Wochen nach Frakturdatum

- Klinische Untersuchung (Ärztliche Behandlung und/ oder Beratung)
- Bildgebende Verfahren nach klinischem Standard der UCH (Rö/CT) / bei medizin. Notwendigkeit
- Erfassung patient/-innenbezogener und medizinischer Angaben
  (allgemeine Angaben,
  Angaben zum Unfall, zur Fraktur sowie ggf. zu Begleitverletzungen,
  Angaben zur (Nach-) Behandlung der Fraktur,
  Angaben zum Zustand vor der Fraktur)
- Ausfüllung von Fragebögen
  (DASH-Score zur Erhebung von Einschränkungen in Arm, Schulter und Hand,
  EQ-5D-Fragebogen zur Erhebung der gesundheitsbezogenen Lebensqualität)

Nachuntersuchung 1: 6 Wochen nach Frakturdatum (bei konservativer Therapie) bzw. OP-Datum (bei operativ erfolgter Behandlung vor Nachuntersuchungstermin 1)

- Klinische Untersuchung
- Bildgebende Verfahren (Rö/CT) nach Standard der UCH / bei medizin. Notwendigkeit
- Erfassung medizinischer Angaben
- Erhebung des Constant-Murley-Fragebogens zur Beurteilung der Schulter-Arm-Funktion
- Foto-/ Videodokumentation der Funktionalität von Schulter und Oberarm
- Ausfüllung von Fragebögen

Nachuntersuchung 2: 12 Wochen (3 Monate) nach Frakturdatum bzw. OP-Datum

- folgende Nachuntersuchungen siehe Vorgehen bei Nachuntersuchung 1

Nachuntersuchung 3: 24 Wochen (6 Monate) nach Frakturdatum bzw. OP-Datum

Nachuntersuchung 4: 52 Wochen (1 Jahr) nach Frakturdatum bzw. OP-Datum

Nachuntersuchung 5: 2 Jahre nach Frakturdatum bzw. OP-Datum

Betreuungsangebot bzw. weitere Nachuntersuchungen im jährlichen Abstand nach Frakturdatum bzw. OP-Datum lebenslang sinnvoll

Alle Nachuntersuchungen finden in der Unfallchirurgischen Poliklinik der MHH statt.

Die Einhaltung Ihrer Nachuntersuchungstermine trägt entscheidend zu der Aussagekraft und dem Erfolg des Registers bei. Sollten Sie bei Ihrer jeweiligen Nachuntersuchung keinen neuen Termin erhalten bzw. vereinbart haben, wenden Sie sich bitte für eine zeitnahe Terminabsprache wochentags (Mo-Do 8:00-15:00 Uhr, Fr 08:00-12:30 Uhr) an die Studienkoordination der Traumastiftung unter der Telefonnummer 0511 / 532 - 8427 oder die Unfallchirurgische Poliklinik unter 0511 / 532 - 2099.

Bitte vergessen Sie nicht, zu Ihrer jeweils ersten Untersuchung eines neu angebrochenen Quartals eine neue Überweisung vom Facharzt für Orthopädie oder Unfallchirurgie mitzubringen.

2. Einverständniserklärung Hannover Humerus Register (HHR)

Name, Vorname des Patienten/ der Patientin: .... | Bitte Kleinen Patientenaufkleber verwenden | ....

Geburtsdatum: ....

Ich erkläre mich bereit, **freiwillig** an der Erhebung meiner krankheitsbezogenen Daten / der krankheitsbezogenen Daten meines Kindes bzw. meiner Betreuungsperson im Rahmen des **Hannover Humerus Registers (HHR)** der Klinik für Unfallchirurgie der Medizinischen Hochschule Hannover teilzunehmen.

Ich bin von Herrn / Frau Dr. med. .... (Name und Stempel verwenden) ausführlich und verständlich über die Ziele und den Verlauf des HHR aufgeklärt worden. Meine freiwillige Teilnahme am HHR kann ich jederzeit und ohne Angabe von Gründen beenden, ohne dass mir dadurch Nachteile für meine weitere medizinische Betreuung und Behandlung entstehen.

Zugleich bin ich damit einverstanden, dass meine im Rahmen der konservativen Erstbehandlung bzw. im Rahmen einer Operation sowie eventueller Folgeoperationen ermittelten klinischen Daten und die im Verlauf nachfolgender Kontrolluntersuchungen erhobenen Informationen zu meinem Oberarm, meinem Gesundheitszustand und meiner gesundheitsbezogenen Lebensqualität mit verschiedenen Fragebögen, elektronisch sowie durch klinische Fotos und / oder Videos dokumentiert werden.
Die Fragebögen wurden mir zur Information übergeben und ihr Inhalt ist mir bekannt. Bezogen auf die Foto- / Videodokumentation weiß ich, dass die von mir angefertigten Aufnahmen als Teil der Behandlungsunterlagen neben der Therapieplanung, Verlaufskontrolle und Qualitätssicherung nach außen hin in anonymisierter Form (unkenntlich machen personenidentifizierender Merkmale wie Gesicht, Tattoo's) u.a. auch für wissenschaftliche Vorträge, Untersuchungen und Veröffentlichungen genutzt werden können.

Meine personenbezogenen Daten können nur von meinem behandelnden Arzt bzw. meiner behandelnden Ärztin, weiteren mit dem Fall betrauten Ärzt/-innen der Klinik für Unfallchirurgie der MHH sowie den Mitarbeitenden der Vertrauensstelle des Registers eingesehen werden und unterliegen der Schweigepflicht. Die Vertrauensstelle des HHR entspricht dabei der Registerstelle und besteht aus ausgewählten Ärzt/-innen der Klinik für Unfallchirurgie der MHH sowie der Studienkoordination der Traumastiftung.

Eine **Kopie der Patient/-inneninformation** sowie dieser unterschriebenen **Einverständnis- und Schweigepflichtsentbindungserklärung** habe ich erhalten und deren Inhalte verstanden. Das Original verbleibt in meiner Patientenakte oder unzugänglich für Dritte bei der Registerstelle des HHR.

Aufgetretene Fragen wurden mir vom Arzt bzw. von der Ärztin verständlich und genügend beantwortet. Ich hatte ausreichend Zeit, mich zu entscheiden. Ich habe zur Zeit keine weiteren Fragen mehr.

Datenschutzrechtliche Informationen HHR

Ich habe verstanden und erkläre mich damit einverstanden, dass meine medizinischen, gesundheits- und lebensqualitätsbezogenen Daten im Rahmen des Hannover Humerus Registers erhoben, in Papierform sowie pseudonymisiert (dies bedeutet mein Name wird durch eine Kennnummer ersetzt ohne Angabe von Namen, Anschrift, Initialen o. ä.) auf elektronischen Datenträgern zusammen mit den von mir angefertigten, noch nicht anonymisierten klinischen Fotoaufnahmen gespeichert, ausgewertet und archiviert werden. Um meine Informationen vor Unbefugten zu schützen, erfolgt die elektronische Übermittlung meiner registerbezogenen Daten für die Speicherung, Sammlung und Auswertung dabei ausschließlich unter Verknüpfung mit dem Pseudonym. Zur Erstellung meines Pseudonyms sowie zur Kontaktaufnahme dürfen meine persönlichen Kontaktdaten von der Registerstelle gespeichert werden. Zugriff auf diese Daten hat ausschließlich autorisiertes bzw. mit der Kontaktaufnahme beauftragtes Personal. Die Daten werden nicht an Dritte weitergegeben. Mir ist bekannt, dass ich jederzeit ein Recht auf Auskunft über meine gespeicherten Daten besitze und fehlerhafte Daten berichtigen lassen kann.

Die externe Verwendung (außerhalb des Projektes) meiner im Register gespeicherten Fotoaufnahmen u. a. für wissenschaftliche Publikationen, Untersuchungen und Vorträge erfolgt ausschließlich in anonymisierter Form. Auch die Weitergabe meiner registerbezogenen Daten an Dritte einschließlich den Zwecken zur wissenschaftlichen Auswertung einer beauftragten Stelle und für Vorträge, Veröffentlichungen sowie Untersuchungen findet nur in pseudonymisierter Form statt. Die Erlaubnis zur Verwendung meiner Fotoaufnahmen im Rahmen des HHR kann ich jederzeit mit Wirkung für die Zukunft widerrufen.

Zum Zwecke einer Originaldatenprüfung (zur Qualitätssicherung und für eine evtl. notwendige gesetzlich geregelte Datenüberprüfung) dürfen bei Bedarf zur Verschwiegenheit verpflichtete Beauftragte Einblicke in meine Patientenakte und -daten nehmen.

Beim Widerruf meiner Einwilligung für die Teilnahme am HHR habe ich das Recht, die Löschung aller meiner bis dahin gespeicherten personenbezogenen Daten zu verlangen. Die Beendigung der Mitwirkung erkläre ich in schriftlicher Form gegenüber meinem behandelnden Arzt bzw. meiner behandelnden Ärztin, oder sollte diese/-r nicht mehr erreichbar sein, gegenüber den Kontaktpersonen der Registerstelle des HHR.

Ich bin damit einverstanden, dass meine Daten nach Beendigung oder Abbruch des HHR archiviert werden. Meine personenbezogenen Daten werden gelöscht, soweit nicht gesetzliche, satzungsmäßige oder vertragliche Aufbewahrungsfristen entgegenstehen. Die übrigen Daten können ohne Rückschlussmöglichkeit auf meine Person (anonymisiert) weiter genutzt werden.

Univ.-Prof. Dr. med. C. Krettek, FRACS
Direktor der Unfallchirurgischen Klinik
Medizinische Hochschule Hannover
Carl-Neuberg-Str. 1 · 30625 Hannover
Tel. 0511 532-2050/2026 · Fax 0511 532-[illegible]
[illegible]

Datum und Unterschrift des Patienten / der Patientin bzw. des gesetzl. Vertreters / der gesetzl. Vertreterin	Datum, Unterschrift und Stempel des verantwortl. Arztes / der verantwortl. Ärztin

3. Schweigepflichtsentbindungserklärung Hannover Humerus Register (HHR)

Ich, ... (Vor-u. Zuname), geboren am ...., wohnhaft .... (Adresse) entbinde alle in die Behandlung meines geschädigten Oberarmes einbezogenen Ärzt/-innen freiwillig von der ärztlichen Schweigepflicht, soweit dies für die Übermittlung meiner für das Hannover Humerus Register (HHR) notwendigen Daten an die autorisierten und zur Verschwiegenheit verpflichteten Beauftragten (Vertrauensstelle des HHR) notwendig ist. Beim Umgang mit meinen Daten werden die Bestimmungen des Datenschutzgesetzes beachtet. Für die Speicherung und Auswertung meiner Daten im Rahmen des HHR wird (mit Ausnahme meiner persönlichen Kontaktdaten) ein Pseudonym verwendet, sodass Unbefugten kein Personenbezug zu den gespeicherten oder ausgewerteten Daten möglich ist.

Ich bin ausdrücklich damit einverstanden, dass die Mitarbeitenden der Vertrauensstelle des HHR und die Ärzt/-innen der Klinik für Unfallchirurgie der MHH meine/-n niedergelassene/-n Ärzt/-innen über meine Teilnahme am HHR informieren können.

Mir ist bekannt, dass ich diese Erklärung über die Entbindung von der Schweigepflicht jederzeit mit Wirkung für die Zukunft widerrufen kann.

....

Datum und Unterschrift des Patienten / der Patientin bzw.
des gesetzlichen Vertreters / der gesetzlichen Vertreterin
